# Extensive marine-terminating ice sheets in Europe from 2.5 million years ago

**DOI:** 10.1126/sciadv.aar8327

**Published:** 2018-06-13

**Authors:** Brice R. Rea, Andrew M. W. Newton, Rachel M. Lamb, Rachel Harding, Grant R. Bigg, Phil Rose, Matteo Spagnolo, Mads Huuse, John M. L. Cater, Stuart Archer, Francis Buckley, Maral Halliyeva, Jane Huuse, David G. Cornwell, Simon H. Brocklehurst, John A. Howell

**Affiliations:** 1 School of Geosciences, University of Aberdeen, Aberdeen, UK.; 2 School of Earth and Environmental Sciences, University of Manchester, Manchester, UK.; 3 School of Natural and Built Environment, Queen’s University Belfast, Belfast, UK.; 4 Department of Geography, University of Sheffield, Sheffield, UK.; 5 Apache North Sea Ltd., Aberdeen, UK.; 6 RPS Ichron, Northwich, Cheshire, UK.; 7 Mærsk Olie og Gas A/S, Copenhagen, Denmark.; 8 Lloyd’s Register, Aberdeen, UK.; 9 St. Margaret’s Road, London, UK.

## Abstract

Geometries of Early Pleistocene [2.58 to 0.78 million years (Ma) ago] ice sheets in northwest Europe are poorly constrained but are required to improve our understanding of past ocean-atmosphere-cryosphere coupling. Ice sheets are believed to have changed in their response to orbital forcing, becoming, from about 1.2 Ma ago, volumetrically larger and longer-lived. We present a multiproxy data set for the North Sea, extending to over a kilometer below the present-day seafloor, which demonstrates spatially extensive glaciation of the basin from the earliest Pleistocene. Ice sheets repeatedly entered the North Sea, south of 60°N, in water depths of up to ~250 m from 2.53 Ma ago and subsequently grounded in the center of the basin, in deeper water, from 1.87 Ma ago. Despite lower global ice volumes, these ice sheets were near comparable in spatial extent to those of the Middle and Late Pleistocene but possibly thinner and moving over slippery (low basal resistance) beds.

## Teaser

Ice sheets repeatedly advanced into the central North Sea, south of ~60°N, from 2.53 Ma ago onward moving over slippery beds with low slung profiles.

## INTRODUCTION

**Fig. 1:**
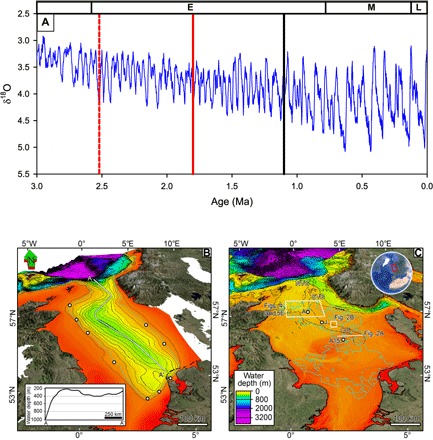
Marine oxygen isotope climate proxy record and modern and Early Pleistocene North Sea bathymetry. (**A**) Composite benthic δ^18^O stack (*42*) climate proxy record, showing the transition from 41-ka obliquity to 100-ka eccentricity forcing between ~1.2 and 0.6 Ma ago. The solid black line indicates the currently accepted timing for major glaciation of the North Sea. The red dashed line shows the earliest glaciation identified from this study, when ice margins were terminating in water depths of ~250 m. The solid red line shows earliest age for grounded ice in deepest part of North Sea, at ~300 m water depths, identified from this study. E, Early Pleistocene; M, Middle Pleistocene; L, Late Pleistocene. (**B**) Base Pleistocene bathymetry shown as depth below modern sea level. The white circles are the fluvial inputs used for the iceberg trajectory modeling, and the light blue line is the −70 m contour representing the Early Pleistocene glacial coastline. This uses the same bathymetric color scale as in (**C**), with contours every 50 m. The inset shows a section along the line A–A′ illustrating the bathymetric lip south of 60°N shown as depth below modern sea level (−70 m for Early Pleistocene glacial maxima). MegaSurvey three-dimensional (3D) seismic data courtesy of PGS. (C) North Sea, present day with the North Viking Graben (NVG), South Viking Graben (SVG), and Central Graben (CG) marked (gray dashed lines). It should be noted that the Norwegian Channel (directly east of NVG and SVG) had not been eroded during the Early Pleistocene timeframe of interest in this paper. The locations of other figures are shown. The white circles are drill sites, and the light blue line delimits the 3D seismic coverage. Bathymetric contours are every 100 m. A, Aviat; J, Josephine; A15, well A15-03.

Northwest (NW) Europe is a critical region for understanding past, present, and future ocean-atmosphere-cryosphere coupling, as it is adjacent to the North Atlantic, where, for example, meltwater inputs can affect the ocean conveyor (*1*) and ice sheet geometries can affect atmospheric circulation (*2*, *3*). Evidence from the North Atlantic ice-rafted detritus (IRD) belt has demonstrated that marine-terminating ice masses were present on Fennoscandia and the British Isles, in the Early Pleistocene (*4*, *5*). To date, the geometry of these early ice sheets is essentially unconstrained, with the exception of the western margin of the Fennoscandian ice sheet (FIS), between 61°N and 67°N (*6*). The Norwegian shelf in this region migrated westward some 150 km through deposition of a series of prograding clinoforms and sediment wedges, from ~2.8 million years (Ma) ago onward. Subsequent erosion of the clinoform topsets from the inner to mid-shelf will have likely removed any evidence for grounded ice and/or ice margins (*6*), precluding determination of ice sheet geometries. The change to high deposition rates and the coarse heterolithic composition of the sediments are consistent with fast-flowing marine-terminating ice margins with high ice flux and erosion potential (*6*). These data suggest a large, but geometrically unconstrained, FIS. The current paradigm is that ice sheets did not enter the center of the North Sea until ~0.7 Ma ago (Fig. 1A) (*7*), after the Middle Pleistocene transition (MPT), although this has recently been challenged (*8*, *9*). Here, we present a detailed and comprehensive regional investigation, using data from seismic reflection surveys and boreholes, on the Early Pleistocene glaciations of the central and southern North Sea. Our work demonstrates that the British-Irish ice sheet (BIIS) and FIS were nearly as extensive in the North Sea before the MPT as those occurring after the MPT, when the climate system changed from a 41-ka (thousand-year) (obliquity) to a 100-ka (eccentricity) periodicity (Fig. 1A), in the absence of an orbital forcing signal. This challenges the idea that full glaciation of the North Sea occurred only after the MPT, when the basin (Fig. 1B) had been infilled and the climate system was responding at the eccentricity frequency (*10*). The presence of spatially extensive Early Pleistocene ice sheets in Europe provides support for the regolith hypothesis, a proposed driver of the MPT, and the Early Pleistocene low-slung ice sheet concept (*11*).

**Fig. 2:**
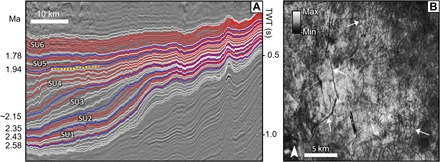
Clinoform geometry, chronology, and iceberg scours. (**A**) Lines show the semiautomatically picked reflectors from the 3D seismic data. Blue lines show the seismic horizons that are used as a dating control and that define the six seismic units (see Methods and fig. S6). The red horizons are those with iceberg scours present, and the white horizons are those where glacial landforms have not been identified. The seismic line location is shown in Fig. 1C. The yellow dotted line shows the stratigraphic location of the surface mapped and displayed in (B). Mapping from picked reflectors/surfaces provides more robust chronological and depth/TWT (two-way travel time) control than mapping from time slices (*22*) (horizontal planes of equal TWT). (**B**) Surface created from the 3D seismic with the root mean square amplitude attribute displayed. White arrows show examples of curvilinear iceberg scours that were mapped on this surface. MegaSurvey 3D seismic data courtesy of PGS.

During the Pliocene to Early Pleistocene, the North Sea was a long (~600 km), narrow (<200 km), and deep (up to ~400 m) marine inlet (Fig. 1B) constructed by the interplay of deltaic to prodeltaic sedimentation and subsidence above Mesozoic graben structures, the Viking and Central grabens (Fig. 1C) (*12*, *13*). It connected to the Atlantic Ocean in the north, but there was no connection through the English Channel to the south (*14*). The basin was the major depocenter through the Plio-Pleistocene, in places accumulating 1.1 to 1.2 km of sediments (*13*), becoming infilled by the Middle Pleistocene (*10*). Rivers entering the basin from the south and east, draining Europe and Scandinavia, dominated the sediment flux. These formed a progradational infill that provides a very high–resolution record of paleoclimatic fluctuations (*15*–*17*), which can be traced northward into the Central and Viking grabens (Fig. 2A) (*13*). Iceberg scours have been reported in the North Sea dating from the earliest Pleistocene (fig. S1 and table S1) and have been interpreted to indicate evidence for ice sheet margins grounded in water hundreds of meters deep (*18*). The iceberg source has tentatively been interpreted to be north of 60°N, which is corroborated by seismic stratigraphic data (*6*). However, iceberg scour mapping has been piecemeal, limited to small spatial scales [for example, (*18*)] and poorly constrained chronologically (fig. S1 and table S1).

## RESULTS

### North Sea geometry, icebergs, and circulation

We present new, detailed subsurface mapping here, from a basin-scale 3D seismic reflection data set (Fig. 1C and fig. S1), calibrated to a robust high-resolution chronostratigraphic record from the southern North Sea (*13*, *16*). The earliest Pleistocene, from 2.58 to 1.78 Ma ago, was divided into five seismic units (SU1 to SU5) with SU6, the topmost, representing the section above 1.78 Ma ago (Fig. 2A and fig. S2). We mapped all regionally continuous reflectors, chronostratigraphically calibrated them, and analyzed them for geomorphological evidence of glaciation (see Methods). More than 8000 individual iceberg scours were identified (fig. S2). SU1 contains three scoured horizons from marine isotope stage (MIS) 100, 98, and 96 (2.53 to 2.44 Ma ago). SU2 contains scours from MIS 94 and 92 (2.40 and 2.36 Ma ago). No scours are correlated with MIS 90, 88, or 86 (2.30 to 2.28/2.24 Ma ago) in SU3; however, evidence may have been removed by sediment reworking due to repeated exposure of the shelf at this time (*17*). SU4 contains scours from MIS 82 to 74 (~2.15 to 1.94 Ma ago), including the two most significant Early Pleistocene sea-level lowstands (MIS 82 and 78) (*19*). As the basin became increasingly terrestrial during lowstands (fig. S2) (*13*, *17*), scours are less common in the southeast. SU5 and SU6 (fig. S2) contain >2000 scours across several surfaces spanning MIS 72 to 64 (1.91 to 1.78 Ma ago), with none present in the south after 1.78 Ma ago.

**Fig. 3:**
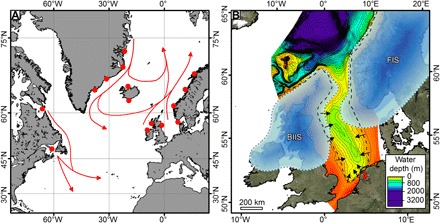
Early Pleistocene pan–North Atlantic modeled iceberg trajectories and inferred ice sheet geometries around the North Sea. (**A**) Schematic showing the modeled iceberg trajectories for all studied release sites (see fig. S3 for individual experiment iceberg density fields). Approximate areas where icebergs were seeded into the Early Pleistocene ocean simulations are shown by the red dots. (**B**) Hypothetical geometries of the BIIS and FIS during the Early Pleistocene from MIS 100 (2.53 Ma ago) with the dashed ice sheet margins indicating that they are unconstrained. The western margin of the FIS, between 61°N and 67°N, is believed to have encroached out toward the shelf break as it prograded westward, but no evidence suitable for constraining the exact ice margin geometry has yet been identified in the earliest Pleistocene section (*6*). The iceberg scours in the North Sea, mapped in this study, required an ice sheet margin/s to have been present south of the bathymetric lip at ~60°N (Fig. 1B), grounded in water depths on the order of 250 m (Fig. 1B, figs. S1 to S3, and table S1). The back dashed line represents the −70-m Early Pleistocene glacial shoreline determined from the seismic data, and the black arrows indicate the freshwater inputs based on modern locations (extended outward to the paleoshoreline) and paleodrainage patterns on continental Europe. Water depths are relative to modern sea level. Compared to previous interpretations, the hypothetical FIS has a greater southward extension, and the BIIS is greatly expanded (*18*, *58*). MegaSurvey 3D seismic data courtesy of PGS.

Two lines of evidence constrain the origin of the icebergs. The first comes from the North Sea circulation and the paleoceanography of the northeast (NE) Atlantic. River discharge into the North Sea, from continental Europe, Scandinavia, and Great Britain, would have forced a buoyant surface current to flow out of the basin, with return inflow at depth. This surface current would have limited the potential for icebergs to enter from the North Atlantic (Figs. 1B and 3A). The paleoceanography of the NE Atlantic appears consistent through time, with northward-moving currents during glacial periods (*20*–*22*). Trajectories of icebergs sourced from western and northern Norway, the western side of the British Isles, Greenland, and North America have been modeled using an intermediate complexity climate model (*21*). We used the Early Pleistocene bathymetry of the North Sea (*13*), combined the current river discharge into the North Sea and Baltic into nine input points (Fig. 1B), and tested various contrasting wind forcing scenarios (see Methods). Icebergs from Greenland, North America, and the western British Isles were found to bypass the entrance to the North Sea and drift northward into the Norwegian Sea, joining those originating from western and northern Norway (Fig. 3A and fig. S3) (*18*). For all the wind forcing scenarios tested, we found that no modeled icebergs entered the North Sea from the Atlantic.

The bathymetry of the North Sea during the Early Pleistocene (SU1) provides the second, independent, line of evidence. The new basin-scale mapping identified a shallow bathymetric lip, south of 60°N, which limited the draft of any North Atlantic icebergs that could have potentially entered the North Sea (Fig. 1B). The TWT differential between the deepest scours in the basin and the lip, derived from 2D seismic data (3D data coverage does not encompass the lip high point), is on the order of at least 65 ms. This difference equates to ~65 m water depth, that is, icebergs creating the deepest scours had greater draft than the lip would permit to pass. Removing the Pleistocene infill and rebounding the basin vertically allow an approximation for the absolute depths (see Methods). Assuming a −70 m eustatic sea-level fall at glacial maximum (*19*, *23*, *24*), the lip water depth would have been ~155 m, but in SU1, iceberg drafts of ~230 m are indicated. Even if, contrary to our modeling results, the NE Atlantic circulation permitted icebergs to be advected into the central and southern North Sea (*18*), the lip would have limited their draft to a depth that was 65 to 75 m too shallow (Fig. 1B) to have formed the deepest scours observed.

In summary, the first iceberg scours are recorded, at the latest, during MIS 100 (Fig. 2A and fig. S2). The modeling and mapping analyses both indicate that the Early Pleistocene icebergs had to be sourced from within the North Sea, requiring ice to have been grounded south of ~60°N, in water depths on the order of at least ~250 m (Figs. 1B and 3B and fig. S2). This redefines the geometry of the NW European ice sheets before the MPT, showing that spatial extents in the Early Pleistocene were near equivalent with those seen in the Middle and Late Pleistocene (*10*).

### Grounded ice in the deep basin

**Fig. 4:**
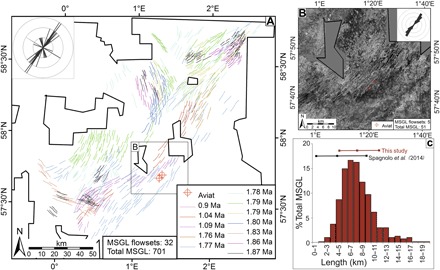
Evidence for grounded ice streams, chronology, and landform metrics. (**A**) Mapped flowsets of MSGLs with color coding indicating estimated minimum ages. The inset rose diagram indicates the average bidirectional orientation of each flowset, and the inset box indicates the location of the mapped surface shown in (B). (**B**) Surface extracted from 3D seismic data, displaying instantaneous amplitude, highlighting MSGLs over Aviat. The inset rose diagram indicates the bidirectional orientation of MSGLs mapped in the flowset. (**C**) All data from the mapped flowsets compare favorably with metrics from a benchmark data set (*27*). MegaSurvey 3D seismic data courtesy of PGS.

Mega-scale glacial lineations (MSGLs) are diagnostic for the presence of grounded ice streams, the drainage arteries of ice sheets (*25*, *26*). Previous mapping has suggested the presence of Early Pleistocene MSGLs, but it was spatially restricted, with poor chronological control (*8*, *9*). The extensive regional mapping from 3D seismic undertaken here has identified multiple paleoseafloor reflectors showing features interpreted as MSGLs with metrics comparable to a global data set (Fig. 4) (*27*). Flowsets of MSGLs are mostly orientated either NW–southeast (SE), or southwest (SW)–NE (Fig. 4, A and B) and are temporally well constrained using seismic ties back to the calibration site in the southern North Sea (see Methods). The MSGLs, paleobathymetry, and chronology show that grounded ice entered the deepest part of the basin (~300-m water depth) initially during MIS 70 (1.87 Ma ago). MSGLs were formed repeatedly between MIS 70 and 22 (1.87 to 0.9 Ma ago), indicating repeated ice stream incursions as the basin was gradually infilled.

**Fig. 5:**
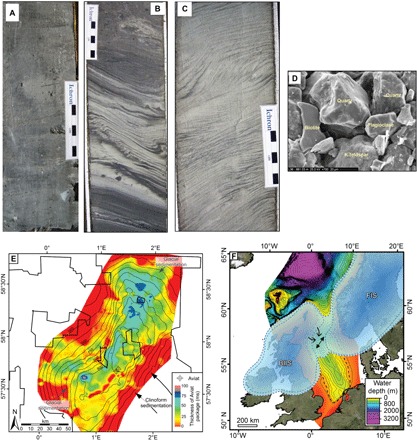
Advance and retreat of grounded ice, sediment flux into the bottom of the basin from 1.87 Ma ago, and possible ice sheet geometries. (**A**) Massive distal glacimarine silty muds with occasional angular clasts interpreted as dropstones (sample location i on fig. S4A). Note that the subhorizontal lines are saw cuts. (**B**) Rippled, very fine sands deposited from hyperpycnal flows during ice advance. They are deformed compressively by subsequent ice stream overriding with the vergence indicating compression from the north/west (sample location ii on fig. S4A). (**C**) Cross-stratified to rippled very fine sand deposited from hyperpycnal flows during ice stream retreat (sample location iii on fig. S4A). Sediment transport appears to be toward the north/west (left), but some cross-sets build toward the right, indicating variable flow directions. (**D**) SEM micrograph illustrating the fresh texture of the grains, noting the lack of solution features (specifically on the feldspars and biotite) or overgrowths (sample location iv on fig. S4A). (**E**) Sediment thickness determined from seismic and borehole log data indicating the extent of the subglacial till. The thickness map is overlaid onto the basin structure at ~1.8 Ma ago. Contours are measured every 50 ms. Sediment flux from the glaciers and the major European rivers (clinoform progradation) are indicated. (**F**) Hypothetical geometries of the BIIS and FIS during the Early Pleistocene from MIS 70 (1.87 Ma ago) onward, indicating near or complete coalescence (the configurations from MIS 100 shown in Fig. 3B are also provided by way of comparison). The dashed ice sheet margin indicates that these limits are unconstrained. The MSGLs directions are indicated by the black arrows, with the single arrowheads indicating the inferred ice flow direction based on paleogeography and paleobathymetry. The dual arrowheads in the center of the basin indicate repeated incursions/ice flows from the NE and SW. The back dashed line represents the −70 m Early Pleistocene glacial shoreline determined from the seismic data. Water depths are relative to modern sea level. Compared to previous interpretations, the hypothetical FIS has a greater southward extension, and the BIIS is greatly expanded (*18*, *58*). MegaSurvey 3D seismic data courtesy of PGS.

Cores recovered from three wells between 878 to 965 m below the seafloor during the exploration and appraisal phases of the Aviat gas field, show a buried, ice-proximal, subaqueous fan (Figs. 1C and 4) (*9*). A basal, massive, distal, glacimarine mud with dropstones (indicating icebergs) (Fig. 5A) is overlain by an ice-proximal sandy-silt unit deposited by meltwater discharging from the margin of an ice stream. These sediments were subsequently deformed (Fig. 5B) (*28*) by an overriding ice stream that deposited a silty-clay deformation till. As the ice stream retreated, it laid down a second ice-proximal sandy-silt unit (Fig. 5C). The stratigraphy is most simply interpreted as a glacial advance-overriding-retreat sequence (fig. S4). The cores come from within the succession containing the SW-NE trending MSGLs noted above (Fig. 4) and confirm the presence of grounded ice in this part of the basin from as early as 1.78 Ma ago (MIS 64).

Analyses show that the cored sediments are dominated by detrital quartz and feldspar with clays accounting for, on average, 22% by weight. Scanning electron microscopy (SEM) micrographs show that feldspars and hornblende are invariably fresh in appearance (Fig. 5D and fig. S4, G and H), indicating that the source ice sheet was actively eroding bedrock, concomitant with genesis by subglacial erosion.

The till identified in the Aviat cores can be mapped from regional seismic, supported by gamma ray, resistivity, and sonic downhole logs. These demonstrate that till covers most of the central part of the basin, in places reaching almost 100-ms TWT (~100 m) thick (Fig. 5E). The seismic unit is associated with unconformities in the SW and NE and thickens downslope from both toward the basin center. This is consistent with marginal till thickening theory (*29*, *30*), indicates sediment flux beneath ice streams repeatedly advancing from both the NE and SW, and is supported by the NE-SW–oriented MSGLs seen ubiquitously in seismic amplitude maps through the till sequence. These data constrain the geometries of the BIIS and FIS from MIS 70 (1.87 Ma ago) onward, showing coalescence/near coalescence in the northern part of the central North Sea.

### Chronology

The regional seismic mapping presented above has been tied chronostratigraphically to borehole data in the southern North Sea and forms the dating framework. Seismic horizons defining the prograding clinoforms in the SE of the central North Sea have been traced back across the shelf and tied into the most detailed Plio-Pleistocene chronology for the North Sea (see Methods) (*16*, *31*). Supporting data come from the Josephine and Aviat wells (Fig. 1B and fig. S6).

## DISCUSSION

The geometries of these early ice sheets, both horizontally and vertically, are important because the Laurentide ice sheet (LIS) and BIIS-FIS affect atmospheric circulation, controlling the trajectory of the polar frontal jet stream (PFJS) (*2*, *3*). The PFJS in turn controls the movement of air masses over the ocean and onto the landmasses, affecting ocean salinity and mixing (*32*), as well as temperature and precipitation patterns on the continents (*33*). Our data show that from MIS 100 (~2.53 Ma ago), there were large icebergs floating in the central and southern North Sea. On the basis of numerical modeling and keel draft limitations, these icebergs had to be sourced from ice sheets grounded within the North Sea, south of ~60°N, in water depths of at least ~250 m (Fig. 3B). Constraining the southern limit of the BIIS-FIS to this latitude is important as this will affect the trajectory of the PFJS and is significantly farther south than had previously been proposed, this early in the Pleistocene (*6*, *18*). This near-complete glaciation of the North Sea occurred some 1 Ma earlier than the previously accepted paradigm (*10*). The till unit, identified from seismic and borehole logs, thickens downslope toward the basin center, and core data and MSGLs are consistent with ice sheets advancing repeatedly into the center of the basin. While MSGLs only provide a bidirectional indicator of ice flow, till unit geometry, core kinematics, Aviat geometry, and the paleogeography and paleobathymetry all indicate that the ice flow has come periodically from both the SW and NE after 1.78 Ma ago (Figs. 4 and 5 and fig. S4). Until now, ice sheet coalescence in the center of the North Sea was believed to have occurred from about 0.7 Ma ago onward, after the infilling of the basin (*7*, *10*). A conjoined BIIS-FIS would have directed the PFJS around the southern margin (*2*, *3*), with implications for climate during the Early Pleistocene in Europe (for example, trajectory of synoptic-scale mid-latitude depression systems and concomitant precipitation patterns) relevant to the interpretation of paleoclimate proxies and to climate modeling.

Such an extensive BIIS and FIS in the North Sea from the beginning of the Pleistocene provide direct support for the low-slung ice sheet concept associated with the LIS (*11*). The Canadian Shield accumulation area of the LIS is very different from the source areas of the BIIS and FIS, which are mountainous and, at the end of the Pliocene, would have been fluvial landscapes characterized by dendritic V-shaped valleys. This mountainous topography in the ice sheet inception regions would have provided high basal resistance to ice flow, resulting in thicker ice over the mountains. Beyond the mountains, the terrestrial landscape was low relief (*34*) and extended much farther into the basin than the present-day shoreline (*13*). This low-relief region was covered by regolith (*34*), such that overlying ice moved under low driving stresses. The yield strength of the sediments covering the shallow marine shelf beyond would have been even lower. These weak soft sediment beds, together with the high basal resistance created by the mountains in the accumulation area, would have given the ice sheets a “Mexican hat” profile (*29*, *35*) at least along the flow paths of the ice streams, which would likely have formed across the coastal lowlands and shallow marine shelf.

A low-slung ice sheet requires either a lower equilibrium line altitude (elevation where annual accumulation equals ablation) or a smaller accumulation area ratio (ratio of accumulation area to total area) in comparison to a thicker ice sheet with the same footprint (fig. S5). This can be achieved by either reducing ablation (lowering air temperatures) or enhancing accumulation (precipitation) or some combination of both. Compared with the Late Pleistocene, air temperatures in the Early Pleistocene appear higher (*36*–*39*), while precipitation estimates are, at best, tentative (*38*, *39*). Assuming that air temperatures were warmer, enhanced precipitation (accumulation) would be required to sustain low-slung ice sheets in the Early Pleistocene, which, given the increased enthalpy, is not unrealistic. This scenario fits best with the concept of the weak bedded low-slung (low driving stress) ice sheets, that is, enhanced accumulation is balanced by the increased ice flux facilitated by a weak substrate. The Mexican hat profiles proposed for the BIIS and FIS partially offset the precipitation enhancement required by elevating the accumulation zone.

Steep accumulation and ablation gradients on the ice sheets, implied from above, generate high ice fluxes and increase the potential for erosion. Southern North Sea borehole data indicate an order-of-magnitude increase in sedimentation rates at the Plio-Pleistocene boundary and higher clay content during MIS 103 to 92 (2.58 to 2.35 Ma ago), after which the gamma ray signal begins to decline (fig. S7) (*15*, *16*). The change in sedimentation rates and clay content suggests that the regolith was rapidly stripped from the landmasses around the North Sea, at least along ice stream flow paths and the main river valleys, within the first quarter of a million years of the Early Pleistocene, as hypothesized for the LIS (*40*). This is supported by the “fresh” mineralogy (Fig. 5D and fig. S4) identified in the cores from Aviat, which indicates that by the time of deposition (1.78 Ma ago) the BIIS and/or FIS had stripped regolith from beneath at least parts of the main ice streams and were eroding bedrock. Despite removal of some of the regolith cover, ice sheets repeatedly extended into the deep basin after MIS 92 facilitated by basin infilling and probably by onshore landscape evolution, that is, parabolic glacial valleys.

It is important at this point to consider the impact of the findings presented above for ongoing research investigating the veracity of the ice volume/sea-level (IV-SL) interpretations from stacked records (*41*) [for example, LR04 (*42*)]. The δ^18^O signal in benthic foraminifera is driven by the preferential accumulation of ^16^O in terrestrial ice sheets (ocean δ^18^O becomes more positive) and changes in ocean bottom water temperature (a 1°C decrease in temperature increases the δ^18^O of precipitated carbonate by +0.25 per mil and is independently derived from the Mg/Ca ratio) (*41*). Both components contribute to the δ^18^O signal, with the contribution from bottom water cooling increasing over time (*24*). The LR04 stack (Fig. 1A) suggests lower amplitude cycles for the Early Pleistocene (smaller δ^18^O shifts), increasing through the MPT and into the large cycles of the Middle and Late Pleistocene. Sources of error in δ^18^O-Mg/Ca–derived IV-SL are mainly related to diagenetic and seawater chemistry changes, such as Mg/Ca ratio. The evidence presented here on the spatial extent of the BIIS and FIS adds to that from North America (*11*, *40*, *43*, *44*) showing that ice sheets in the beginning of the Pleistocene, at glacial maxima, were near equivalent in spatial extent to those of the Last Glacial Maximum. The presence of these large ice sheets still has to be reconciled with the contrasting evidence from the δ^18^O record, and the regolith hypothesis provides one explanation. It remains to be seen how the LR04 (Fig. 1A) and other records may change when diagenetic and secular changes in seawater chemistry effects are accounted for (*41*). Any corrections that shift the δ^18^O signal to more positive values at glacial maxima or reduce the contribution from bottom water cooling (ascribing more of the signal to ice volume change) would be in keeping with the terrestrial evidence of ice sheet extent in North America and Europe.

## CONCLUSIONS

Evolution of Earth’s climate response to orbital forcing during the Pleistocene is intriguing and complex (*4*, *11*, *43*). Feedback between landscape, ice sheets, and climate (*45*) has, for a long time, been ignored, but the potential significance is embodied in the regolith hypothesis (*11*, *43*), which provides a plausible explanation of the MPT (*4*, *11*, *43*, *45*, *46*). The hypothesis is tied to the LIS by virtue of its dominant volumetric contribution to the oceanic δ^18^O signal and evidence of Early Pleistocene southern maxima (*40*, *47*). For the hypothesis to have validity, other Early Pleistocene ice sheets should also be spatially extensive. It has been demonstrated, using IRD, that marine-terminating ice masses were present on Greenland, Fennoscandia, and the British Isles from the end of the Pliocene (*4*–*6*) and in Siberia-Kamchatka and Alaska from the beginning of the Pleistocene (*48*, *49*). To date, constraints on the geometries of any of these ice sheets have been missing, and the presence of distal IRD deposits provides only minimal constraint on ice geometries. Until now, the only supporting evidence of expanded Early Pleistocene ice cover came from the Cordilleran ice sheet (*44*) at 2.64 Ma ago. A regolith cover, similar to that hypothesized for the LIS, has long been known for NW Europe (*34*, *50*, *51*), but definitive evidence constraining the extent of Early Pleistocene ice sheets in this region was missing. This study has integrated 3D seismic stratigraphy and geomorphology, climate modeling, and core and wireline log data to provide conclusive evidence for grounded ice sheets in the northern and central North Sea from MIS 100 (2.53 Ma ago), becoming coalescent by at least 1.78 Ma ago. It provides spatial constraint on the ice sheet geometries and identifies at least one ice margin, demonstrating that these Early Pleistocene ice sheets were comparable in horizontal extent to those seen in the Middle and Late Pleistocene (Figs. 3B and 5F). The geometries of these early ice sheets have important implications for understanding atmospheric and oceanic circulation and climate over the continents. The evidence presented above illustrates the potential for continental shelf sedimentary archives to elucidate the complex, evolving, three-way interaction between ice sheets, orbital forcing, and landscape evolution, a phenomenon only observed, to date, in numerical models (*45*).

## METHODS

### Three-dimensional seismic data and mapping

The 3D seismic volumes used here were from the PGS Southern North Sea MegaSurvey and the PGS Central North Sea MegaSurvey, covering an area of ~128,000 km^2^. The seismic data have a 50 m × 50 m lateral resolution and 10 to 15 m vertical resolution, with an approximately 2 m detection limit, and were used to map multiple key surfaces throughout the surveys. These surfaces were selected on the basis of their seismic geomorphology and sequence-stratigraphic significance (*16*). In addition to manually picked horizons, a semiautomated approach was used to map every reflection within the 3D seismic volume to create a geomodel, which attributes a relative geological age to each surface (*52*). The interpreter can then modify and refine the horizon picks in the geomodel so that it is geologically consistent and accurate. From the completed geomodel, a horizon stack was created, allowing the interpreter to scroll through the basin fill in a similar manner to 3D seismic time slices. Rather than cross-cutting the clinoform stratigraphy of the North Sea, the horizon stacks honor the geological structure, that is, in this instance, the paleoland/seabed surfaces.

The basin-scale seismic stratigraphic framework and relative chronology were calibrated to five absolute ages (2.58, 2.43, ~2.15, 1.94, and 1.78 Ma ago) dated using magnetostratigraphy and an additional age, ~2.35 Ma ago, dated using palynology correlated to the global oxygen isotope curve in borehole A15-03 in the southern North Sea (*31*). Corroboration comes from the Josephine and Aviat wells (22/07a-5Z and 22/07a-6Z) in the central North Sea (Fig. 1B and fig. S6) (*13*, *16*). In this way, chronological control was transferred across the basin along paleosurfaces, mapped at 50 m × 50 m resolution, that bound stratigraphic units spanning the Gelasian (2.58 to 1.78 Ma ago) (fig. S2G). In some areas, the Gelasian is up to ~700 m thick, providing an expanded record of the glacial-interglacial cycles (fig. S6) (*16*).

TWT structure, amplitude, root mean square amplitude, and variance attributes were extracted onto the paleosurfaces. These were investigated using multiple illumination angles to identify glacial landforms. Identification of iceberg scours was aided using morphometric comparison with analog examples of horizontal planforms and vertical seismic profiles of iceberg scours identified elsewhere (*18*, *22*).

Iceberg scours are typically linear and curvilinear in planform with either U- or V-shaped furrows below the general level of the paleoseafloor. The interpretation of vertical features is often limited by the seismic resolution so the analyses are predominantly undertaken on the planform metrics mapped from the paleoseafloors. To determine which features should be interpreted as iceberg scours, rather than artifacts from the data acquisition or faults related to salt tectonics, careful analyses of the horizons and their attributes were undertaken by the interpreters. A further caveat to interpreting scours in the geological record is whether these features were caused by iceberg keels or sea ice pressure ridges. Pressure ridge keels rarely reach drafts greater than 40 to 55 m deep or widths >10 m (*18*, *53*, *54*). All curvilinear and linear features close to the paleoshelf break, where water depths are typically 100 to 200 m, were therefore interpreted as iceberg scours.

When scours were identified, they were digitized by drawing a polyline along the central axis of the scour. The morphometric characteristics show typical lengths and widths of 2 to 5 km and 50 to 250 m, respectively, consistent with iceberg genesis. Iceberg-scoured surfaces were constrained to specific seismic units within the chronostratigraphic framework and were then qualitatively compared to records of IRD (*55*, *56*) in the North Atlantic to provide an impression of which glacial stages had higher levels of IRD and presumably increased iceberg production (fig. S2G).

Mapping of MSGLs followed a similar methodology to that for iceberg scours described above. MSGLs are typically observed as multiple ridge-furrow features 1 to 10 km long and 100 to 1000 m wide and were identified on the basis of their characteristic morphology (*27*), from amplitude and root mean square amplitude extractions of the paleosubglacial bed. The thalweg of each lineation was digitized as a single polyline. MSGLs were then classified into separate flowsets (*57*) according to the estimated age of formation (fig. S6), location, and orientation. Length and orientation metrics were determined automatically from the polylines, and the average orientation for each flowset was calculated (Fig. 4).

### Sediment backstripping and crustal rebound

To generate a depth estimate of the paleobathymetry in the North Sea, we followed a backstripping approach because of the difficulty in fully reconstructing the seafloor at the start of the Pleistocene from primary paleoenvironmental proxies that are not available. For example, the near coastal areas of the paleobasin have been removed by later glacial, subaerial, and marine erosion cycles during the Pleistocene, thus leaving a heavily fragmented record of paleoshorelines, although clinoform breakpoints and iceberg-scoured shelf areas provide some constraints on paleowater depths. To generate an estimate of the base Pleistocene bathymetry (2.58 Ma ago), we mapped the entire Pleistocene sedimentary succession in the North Sea and along the mid-Norwegian margin, including the North Sea Fan, which is a key area for determining iceberg flux into the North Sea.

The thickness maps of the glacial succession (*6*, *13*, *58*) were used to estimate the volume of Quaternary sediment to be removed from the basin. The sediment thickness maps from the North Sea (*13*, *55*) were already depth-converted, but the thickness map for the mid-Norwegian margin was not. Using acoustic velocities of 1700 to 1900 m s^−1^ (*58*), the thickness map (*13*) was converted from time to depth. The thickness maps were then georeferenced in ArcMap and manually blended using digitized contours to form a continuous record of the Quaternary succession. The thickness map was then removed from the contemporary General Bathymetric Chart of the Oceans bathymetry (Fig. 1B) to generate the “base Pleistocene” surface.

Isostatic compensation followed a two-stage approach. First, a profile was taken across the basin, and a number of stratigraphic surfaces were iteratively decompacted and rebounded (*13*) using the backstripping and Airy Isostasy methods (*59*). The ratio between the 2D profile depth and the sediment thickness was determined as 0.36, which provided a simple rebound calculation tool (for example, sediment thickness × 0.36). That is, if the sedimentary deposit was 1000 m, then the base Pleistocene seafloor was rebounded to 360 m below sea level. This was tested against a large number of points and proved satisfactory for our purposes.

The correction was then applied to the base glacial surface. The result was checked to ensure that it had generated a geometry that was geologically plausible, compatible with our mapping and the literature. This simplified method is preferred because of lack of constraints on the ice sheet thicknesses, loading duration, and location of the shorelines, so we ignored the effects of glacio-isostatic crustal depression and ice sheet gravity effects. For similar reasons, it does not account for lithospheric flexure. This provides a reasonable first-order estimate of the paleobathymetry at the beginning of the Pleistocene. This surface provided the basis for iceberg draft calculations and was also used as an input for the iceberg trajectory modeling described below.

### Iceberg trajectory modeling

The FRUGAL (Fine Resolution Greenland and Labrador Ocean Model) intermediate complexity climate model (*20*) was set up for Early Pleistocene boundary conditions using the basic approach of the Pliocene Model Intercomparison Project (PlioMIP) (*60*). The model’s orbital parameters were taken as for present, and atmospheric CO_2_ concentration was set to 400 parts per million. The basic land topography and ice mask were those supplied as part of PlioMIP 2, with the sea level set to 70 m below present, as typical of glacial periods during the Early Pleistocene (*19*, *23*, *24*). The topography was modified to remove any sea routes through the Canadian Archipelago, Bering Strait, and the English Channel and revised in the vicinity of the North Sea (Fig. 1C). The basic curvilinear climate model dynamics, thermodynamics, and coupling between ocean, sea ice, icebergs, and atmosphere have previously been described (*21*), but here we use a finer version of the model (*61*), with a resolution varying from 20 km near the coast of Greenland, to 2° × 1.5° in the Southern Ocean. The iceberg module of the model is not, in this study, providing feedbacks to the ocean and atmosphere. Icebergs are, however, forced to move and melt in accordance with the iceberg model’s physics (*62*). The basic state of the model used present-day winds within the energy and moisture balance atmospheric model, as being better suited to the limited Northern Hemisphere ice extent of the PlioMIP 2 boundary conditions (*61*), and was spun up from present-day ocean climatology (*60*) for 210 years. While the deep ocean is still equilibrating at that time, the interest here is in sensitivity to surface and near-surface flows and resulting iceberg trajectories. Upper ocean properties are stable by the end of the spin-up. A companion simulation was run with typical Pleistocene glacial period winds (*21*) for comparison, but the ocean circulation was sufficiently different from that using present-day winds for it to be extremely unlikely, in the glacial simulation, that icebergs from west of the European margin would be able to even approach the BIIS margin, let alone enter the North Sea. Therefore, all experiments reported here, testing the sensitivity of iceberg trajectories to the origin and size of icebergs, used present-day wind forcing.

To maximize the possibility of icebergs seeded at sites away from the European margin reaching and entering the North Sea, only large icebergs of size 5 (500 m × 333 m × 300 m) and 10 (1500 m × 1000 m 300 m) (*62*) were released into the North Atlantic directly. Sensitivity experiments were performed with these releases from the following: (i) eastern Greenland, as is consistent with the PlioMIP 2 ice mask; (ii) key North American sites of Hudson Strait and Gulf of St. Lawrence; (iii) the western Scottish Atlantic coastline; and (iv) western Norway (Fig. 3A). In addition, smaller icebergs of sizes 1 (100 m × 67 m × 67 m), 3 (300 m × 200 m × 200 m), and 5, consistent with the North Sea ice sheet reconstructions discussed here, were released from likely glaciated sites in the northern North Sea (Fig. 3A); this test also had freshwater river discharge entering the North Sea (see Fig. 3C) equivalent to today (~870 km^3^ a^−1^/27,588 cumecs) (*63*, *64*) at inputs related to modern or paleogeographical locations where data were available. The results of these experiments are shown in fig. S4 with a summary schematic shown in Fig. 3A.

### Core sedimentology

Aviat cores were described as they were split, with both cut halves being consulted where necessary. A purely descriptive lithofacies scheme, which defines lithology and sedimentary structures without inferring depositional setting, was used. Subsequently, the depositional model for the cored interval was developed, taking account of all cored intervals, regional seismic interpretations, and in situ and offset well data. Well 22/7a-6Z is deviated, so the core has been used to provide generalized directions of water flow, from ripple climbing directions, and glaciotectonic kinematics, from folds and faults.

### Core mineralogy

Mineralogical descriptions on Aviat core samples were undertaken both visually (thin sections and SEM) and analytically [x-ray diffraction and energy-dispersive x-ray (EDX)] throughout the cored intervals. The combination of mineralogy and texture from the EDX-SEM analyses provided the assessment of grain freshness, interpretation of the grain history, and by default relative age of the weathering-susceptible minerals.

### Biostratigraphy

Volumetric micropaleontological analyses on Aviat core samples were undertaken, applying a uniform and consistent approach to microfossil picking and counting. For all samples, a standard volume was processed, with known splits picked for microfossils. For faunal comparisons, all foraminifera were picked before normalizing back to unity.

The palynological analysis involved a count of the dominant palynomorphs with a subsequent scanning of the slide for additional taxa. A counting method was applied, which attempted to overcome the “blanketing” effect of dominant taxa. The palynomorph count was undertaken on both the >30 μm and the 10 to 30 μm sieve fraction slides with all taxa being recorded, including their relative abundances. Nannofossil counting was conducted along a measured traverse within a concentrated smear slide. The length of the traverse is variable depending on fossil recovery. Following completion of counting, the slide was scanned to detect the presence of rare marker species not previously identified. Individual species counts were normalized to a standard traverse length of 50 mm (≡300 fields of view) to accurately represent variations in the absolute abundance of each species. None of the micro- and nanopaleontological and palynological data were used for paleoenvironmental interpretation, although water depth estimations accord with those determined from the backstripped rebounded crust and eustatic sea-level curve (*19*, *23*, *24*).

### Ice sheet geometries

Hypothetical geometries of the BIIS and FIS are presented in Figs. 3B and 5F. In both figures, the ice margins are marked as dashed lines, as standard for inferred boundaries. Ice margins in the North Sea and along the western Norwegian shelf are better constrained than the remainder of the ice sheets. Data presented above required ice margins of either the BIIS, the FIS, or both to be periodically present in the North Sea, south of ~60°N in water depths of ~250 m from 2.53 Ma ago. Also, the western margin of the FIS had to be proximal to the shelf, periodically, from 2.8 Ma ago between 61°N and 67°N and periodically, on the shelf from ~2 Ma ago (*6*). Subsequently, both the BIIS and FIS were periodically present in the center of the North Sea basin from 1.87 Ma ago, and most likely coalescent (Fig. 5F). On the basis of the evidence presented above, Figs. 3B and 5F show that the FIS is considerably farther south, and the BIIS is very much larger than any previous suggestions (*18*, *58*). However, the exact location of the ice sheet margins remains tentative, and nowhere has any ice margin been absolutely constrained during the timeframe under consideration here, although an ice margin west of, but proximal to, Aviat has been suggested (*9*). The inferred boundaries for the remainder of the BIIS seem comparable with numerical modeling and empirical studies for the last glaciation, which suggests that it is highly dynamic (*65*) at time scales much less than 100 ka. By inference, the FIS was likely also dynamic during the 41-ka cycles of the Early Pleistocene. Further investigations using 3D seismic data volumes, ice sheet, and landscape evolution modeling studies will hopefully provide better constraint on the ice sheet geometries in the future.

## Supplementary Material

http://advances.sciencemag.org/cgi/content/full/4/6/eaar8327/DC1

## SUPPLEMENTARY MATERIALS

Supplementary material for this article is available at http://advances.sciencemag.org/cgi/content/full/4/6/eaar8327/DC1 fig. S1. The modern North Sea, bathymetry, classification, seismic data coverage, well locations, and sites of reported Early Pleistocene iceberg scours. fig. S2. Temporal and spatial patterns of iceberg scours and their relationship with IRD and sea level. fig. S3. Iceberg trajectory modeling experiments. fig. S4. Core stratigraphy, images, and interpretation. fig. S5. Theoretical ice sheet surface profiles demonstrating the effects of basal resistance and topography. fig. S6. A section showing the basis for the seismic stratigraphic framework tied to the magnetic reversal and palynology-based chronology from A15-03 in the Dutch sector of the North Sea (blue lines), which is corroborated by ages from Josephine and Aviat in the UK sector (*16*). fig. S7. Downhole gamma ray logs from the Dutch sector of the southern North Sea. table S1. Details for sites where Early Pleistocene iceberg scours have previously been identified. table S2. Summary information for the biostratigraphical evaluation of Aviat cores 22-7a-5z and 22-7a-6z. References (*66*–*71*)
