# Supplementary material for: Extensive marine-terminating ice sheets in Europe from 2.5 million years ago
Source: Sci Adv. 2018 Jun 13;4(6):eaar8327. doi: 10.1126/sciadv.aar8327 (PMC6007157; doi:10.1126/sciadv.aar8327)
Supplement: http://advances.sciencemag.org/cgi/content/full/4/6/eaar8327/DC1 [file supp_4_6_eaar8327__index.html]

Science Advances | Science Advances

## Supplementary Materials

**This PDF file includes:**

- fig. S1. The modern North Sea, bathymetry, classification, seismic data coverage, well locations, and sites of reported Early Pleistocene iceberg scours.
- fig. S2. Temporal and spatial patterns of iceberg scours and their relationship with IRD and sea level.
- fig. S3. Iceberg trajectory modeling experiments.
- fig. S4. Core stratigraphy, images, and interpretation.
- fig. S5. Theoretical ice sheet surface profiles demonstrating the effects of basal resistance and topography.
- fig. S6. A section showing the basis for the seismic stratigraphic framework tied to the magnetic reversal and palynology-based chronology from A15-03 in the Dutch sector of the North Sea (blue lines), which is corroborated by ages from Josephine and Aviat in the UK sector (*16*).
- fig. S7. Downhole gamma ray logs from the Dutch sector of the southern North Sea.
- table S1. Details for sites where Early Pleistocene iceberg scours have previously been identified.
- table S2. Summary information for the biostratigraphical evaluation of Aviat cores 22-7a-5z and 22-7a-6z.
- References (*66–71*)

Download PDF

**Files in this Data Supplement:**

- Adobe PDF - aar8327\_SM.pdf
