# Supplementary material for: Extensive marine-terminating ice sheets in Europe from 2.5 million years ago
Source: Sci Adv. 2018 Jun 13;4(6):eaar8327. doi: 10.1126/sciadv.aar8327 (PMC6007157; doi:10.1126/sciadv.aar8327)
Supplement: http://advances.sciencemag.org/cgi/content/full/4/6/eaar8327/DC1 [file aar8327_SM.pdf]

Science Advances

AAAS

advances.sciencemag.org/cgi/content/full/4/6/eaar8327/DC1

Supplementary Materials for

Extensive marine-terminating ice sheets in Europe from 2.5 million years ago

Brice R. Rea, Andrew M. W. Newton, Rachel M. Lamb, Rachel Harding, Grant R. Bigg, Phil Rose, Matteo Spagnolo, Mads Huuse, John M. L. Cater, Stuart Archer, Francis Buckley, Maral Halliyeva, Jane Huuse, David G. Cornwell, Simon H. Brocklehurst, John A. Howell

Published 13 June 2018, *Sci. Adv.* **4**, eaar8327 (2018)
DOI: 10.1126/sciadv.aar8327

This PDF file includes:

- fig. S1. The modern North Sea, bathymetry, classification, seismic data coverage, well locations, and sites of reported Early Pleistocene iceberg scours.
- fig. S2. Temporal and spatial patterns of iceberg scours and their relationship with IRD and sea level.
- fig. S3. Iceberg trajectory modeling experiments.
- fig. S4. Core stratigraphy, images, and interpretation.
- fig. S5. Theoretical ice sheet surface profiles demonstrating the effects of basal resistance and topography.
- fig. S6. A section showing the basis for the seismic stratigraphic framework tied to the magnetic reversal and palynology-based chronology from A15-03 in the Dutch sector of the North Sea (blue lines), which is corroborated by ages from Josephine and Aviat in the UK sector (*16*).
- fig. S7. Downhole gamma ray logs from the Dutch sector of the southern North Sea.
- table S1. Details for sites where Early Pleistocene iceberg scours have previously been identified.
- table S2. Summary information for the biostratigraphical evaluation of Aviat cores 22-7a-5z and 22-7a-6z.
- References (*66–71*)

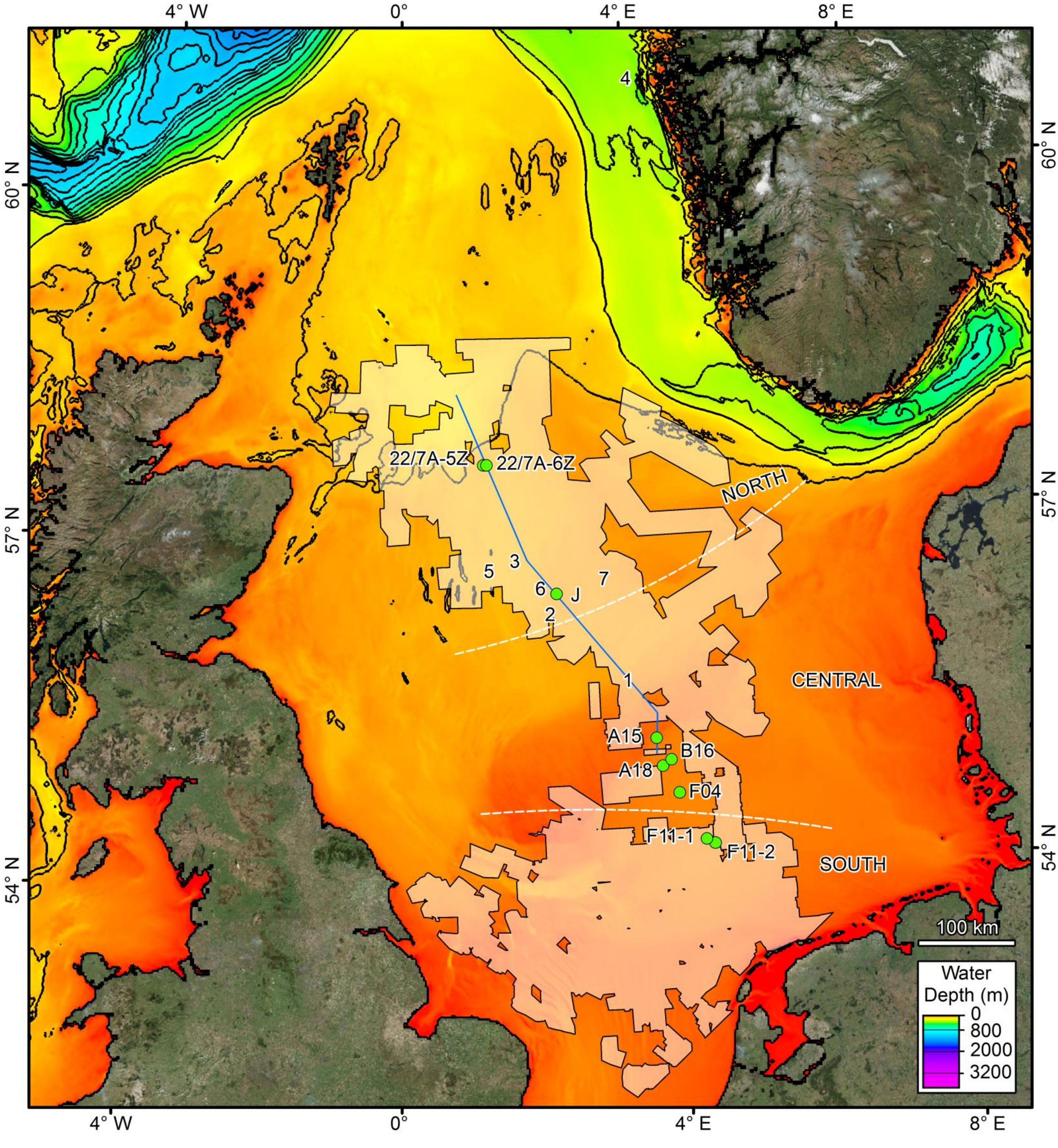

fig. S1. The modern North Sea, bathymetry, classification, seismic data coverage, well locations, and sites of reported Early Pleistocene iceberg scours. The Northern, Central and Southern North Sea are delineated by the white dashed lines. The light shaded polygons show the extent of the regional MegaSurvey 3D seismic data curtesy of PGS. The labelled green circles refer to boreholes mentioned in the text: 22/7A-5Z and 22/7A-6Z are related to Aviat (fig. S4) and those in the Central and Southern North Sea are those plotted on fig. S6. The black numbers (1–7) indicate locations of sites where iceberg scours have previously been observed in 3D seismic data where ages have been assigned, by the authors, to the Early Pleistocene (table S1). Contours are measured every 100 m. The blue line shows the location of the seismic profile display in fig. S6.

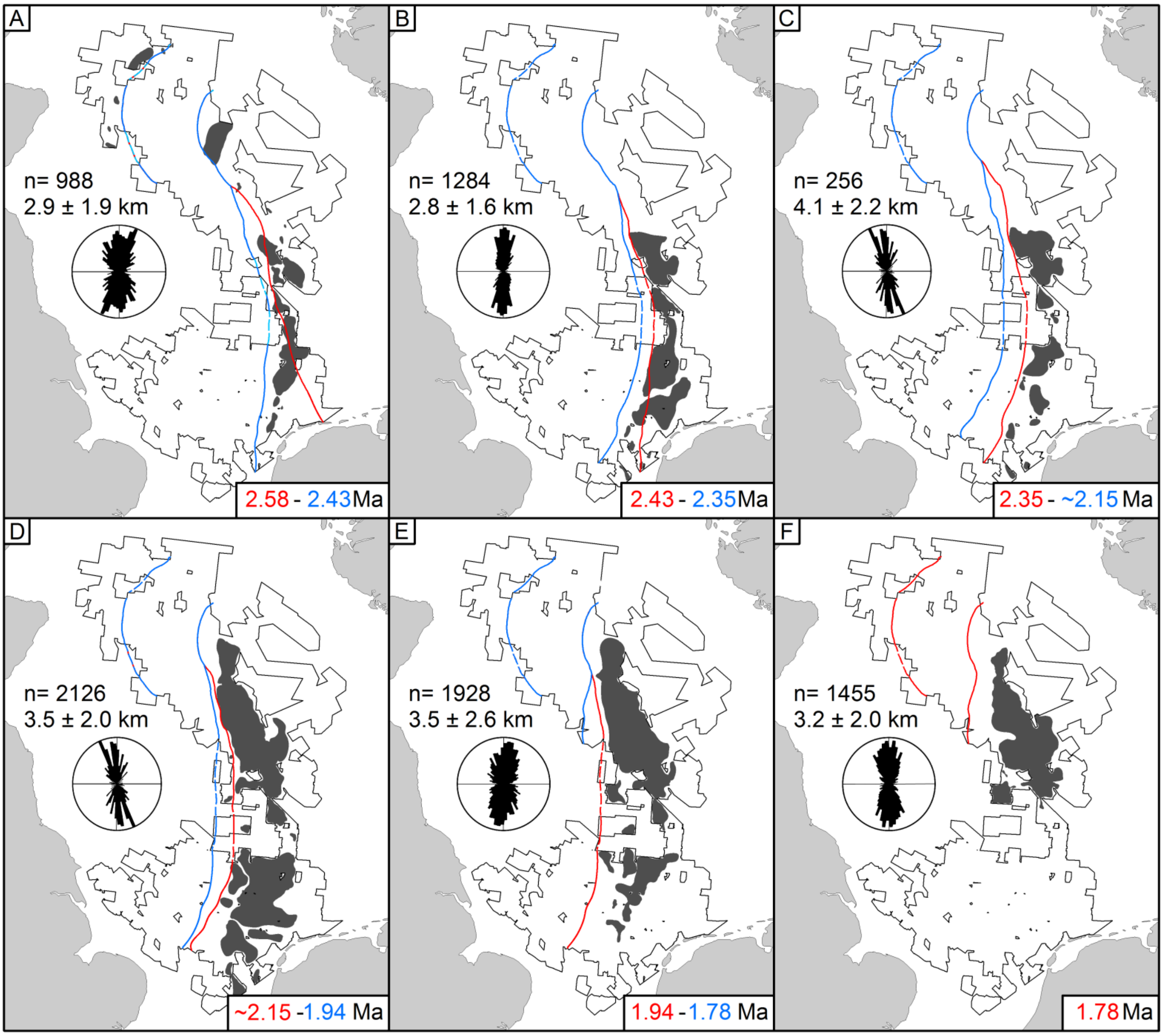
A
n= 988
2.9 ± 1.9 km
2.58 - 2.43Ma
B
n= 1284
2.8 ± 1.6 km
2.43 - 2.35Ma
C
n= 256
4.1 ± 2.2 km
2.35 - ~2.15 Ma
D
n= 2126
3.5 ± 2.0 km
~2.15 -1.94 Ma
E
n= 1928
3.5 ± 2.6 km
1.94 - 1.78 Ma
F
n= 1455
3.2 ± 2.0 km
1.78 Ma

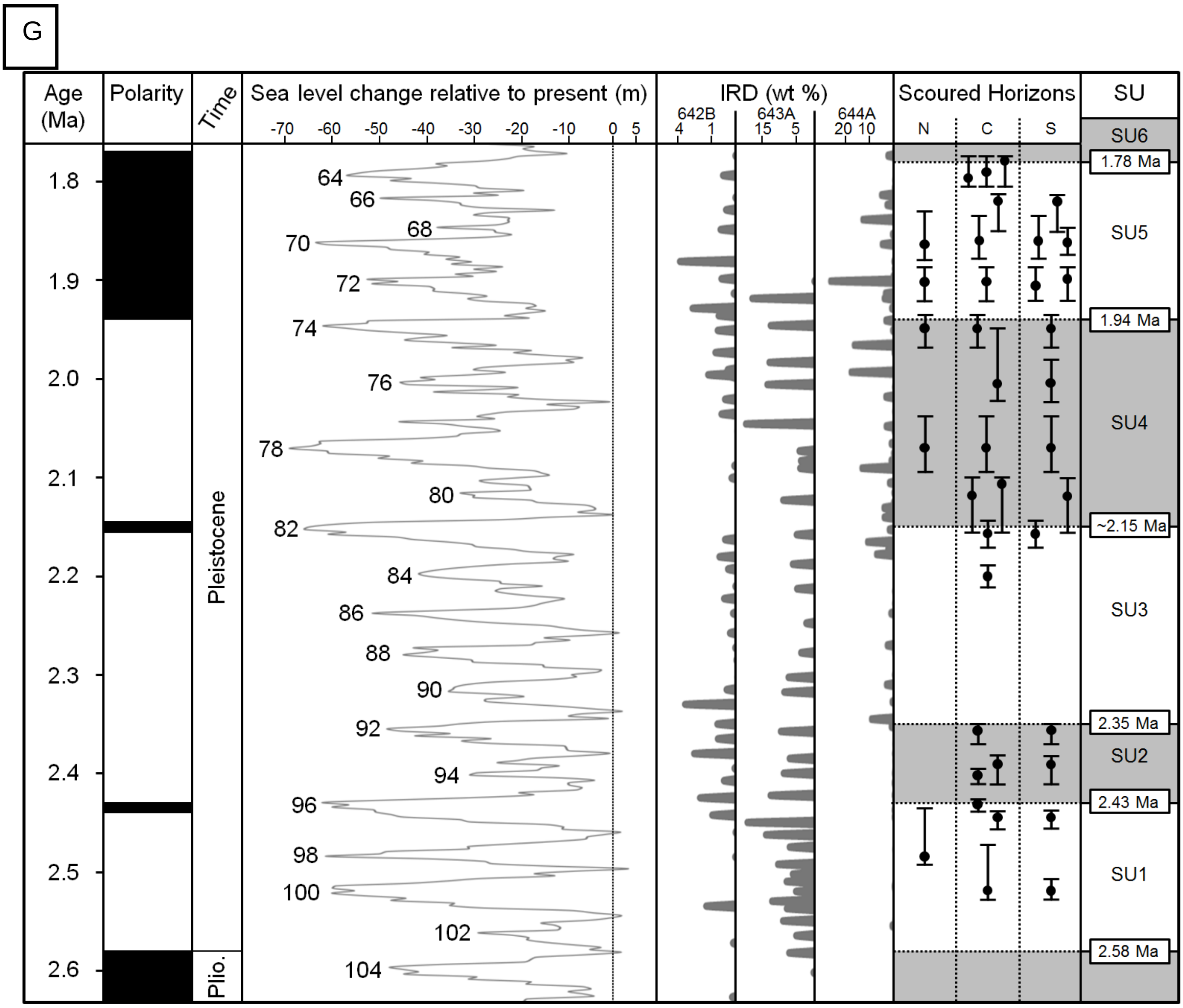
G
Age (Ma)
Polarity
Time
Sea level change relative to present (m)
-70 -60 -50 -40 -30 -20 -10 0 5
IRD (wt %)
642B
4 1
643A
15 5
644A
20 10
Scoured Horizons
N C S
SU
1.8
1.9
2.0
2.1
2.2
2.3
2.4
2.5
2.6
Pleistocene
Plio.
64 66 68 70 72 74 76 78 80 82 84 86 88 90 92 94 96 98 100 102 104
SU6
1.78 Ma
SU5
1.94 Ma
SU4
~2.15 Ma
SU3
2.35 Ma
SU2
2.43 Ma
SU1
2.58 Ma

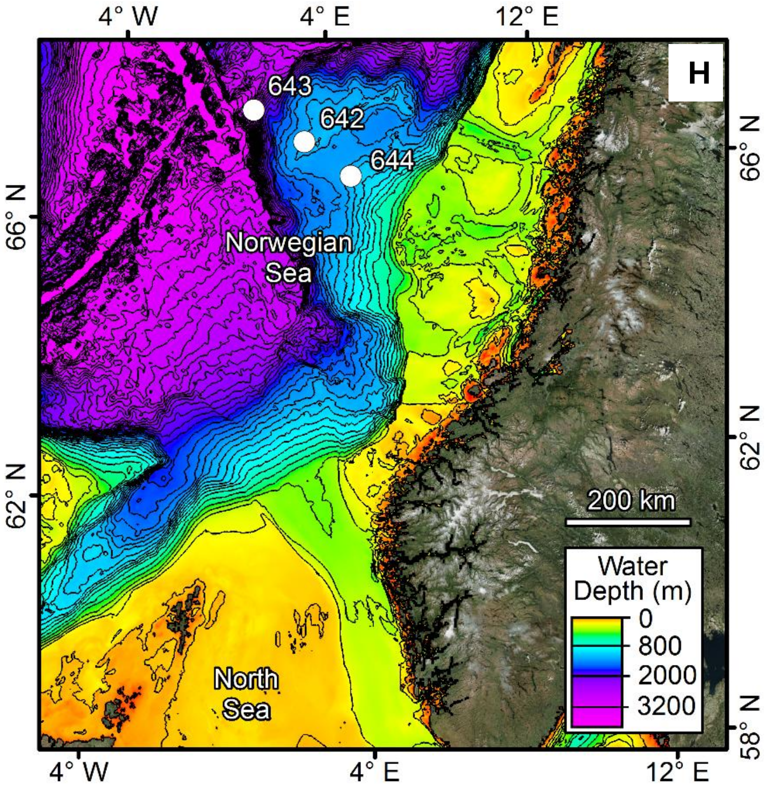
H
4° W
4° E
12° E
66° N
62° N
58° N
643
642
644
Norwegian Sea
North Sea
200 km
Water Depth (m)
0
800
2000
3200

fig. S2. Temporal and spatial patterns of iceberg scours and their relationship with IRD and sea level. Solid black polygons show the areal distribution of iceberg scours during deposition of each seismic unit SU (see Fig. 2A). The location of the shelf edge in each figure is indicated for the start (red line) and the end (blue line) of each time window. This shows how the basin shape changed with migration of the shelf edge towards the west and the reduction in the size of the basin. Note that in the north the blue line sits atop the red line, which is due to minimal change across the full time window of investigation. The southwestern basin shape is not defined because there is no depositional system from which to pick a shelf edge and seismic resolution and post-depositional adjustments preclude interpretation. The numbers of scours mapped within each SU are shown and plotted on rose diagrams, showing a predominantly north to south trajectory. Dashed lines show the shelf edge position outside the 3D coverage determined from 2D seismic profiles. (**A**) deposition of the SU1 unit from 2.58 – 2.43 Ma ago. (**B**) SU2 unit from 2.43 – 2.35 Ma ago. (**C**) SU3 unit from 2.35 – ~2.15 Ma ago. (**D**) SU4 unit from ~2.15 – 1.94 Ma ago. (**E**) SU5 unit from 1.94 – 1.78 Ma ago. (**F**) SU6 unit after 1.78 Ma ago. (**G**) Composite showing the late Pliocene and Early Pleistocene sea level record (*19*) calculated from the global benthic $\delta^{18}O$ record (*42*), with the ice-rafted detritus record (IRD) from ODP Sites 642–644 on the Vøring Plateau (*55*, *56*). IRD abundance is dated using a constant sedimentation rate between magnetically dated intervals. Using the six dated seismic units as a chronological constraint, the scoured horizons are dated by using their position within the seismic unit and by correlations with the sea level record, IRD, and seismic stratigraphy. The black circle indicates the best estimate for the age of the scoured surface and an error range is estimated from the combination of techniques described in methods. See fig. S1 for the location of the northern (N), central (C) and southern (S) areas used to describe iceberg scour locations. (**H**) Location map showing the ODP core sites used for the IRD signal in **G**. MegaSurvey 3D seismic data curtesy of PGS.

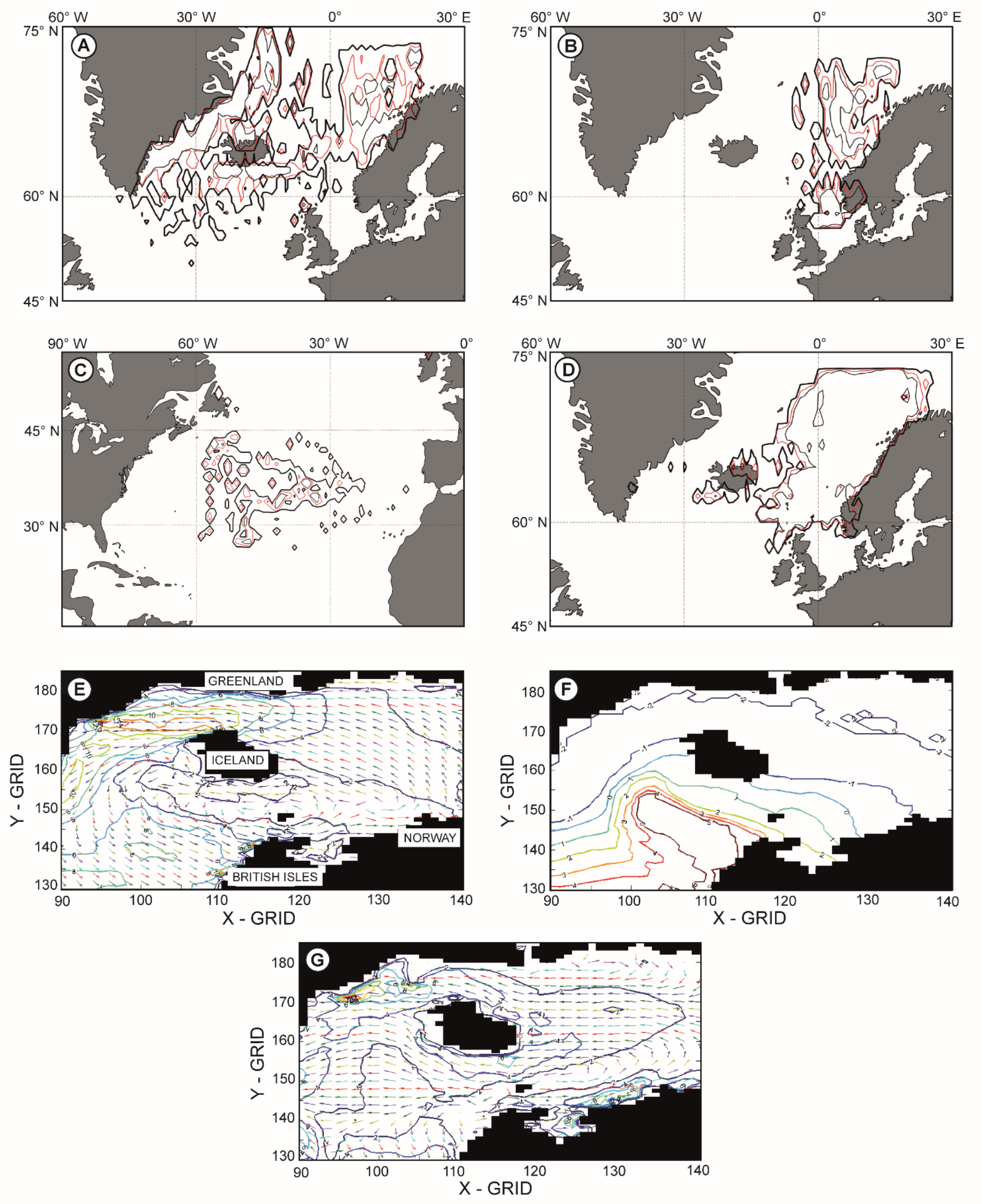

fig. S3. Iceberg trajectory modeling experiments. Iceberg density from 10 year simulations, sampled every 10 days, with icebergs sourced from: (**A**), east Greenland. (**B**) the North Sea. (**C**) North America. (**D**) west Scotland and the Norwegian margin of the Norwegian Sea. Contours are 1 (bold black), 5 (red) and 10 (thin black) icebergs per 1˚x1˚. Note that the densities have been interpolated from the curvi-linear model grid to a regular grid (why, in places, some iceberg density contours impinge on the present day landmasses) and the 10 day sampling gives small gaps in the record where iceberg motion is rapid. (**E**) The present-day-wind simulation forcing surface currents. (**F**) sea surface temperature. (**G**) the glacial-wind simulation forcing surface

currents. E–G are shown on the curvi-linear model grid, with a NE Atlantic excerpt. The surface current contours are in cms^{-1} and the sea surface temperature in $^{\circ}C$.

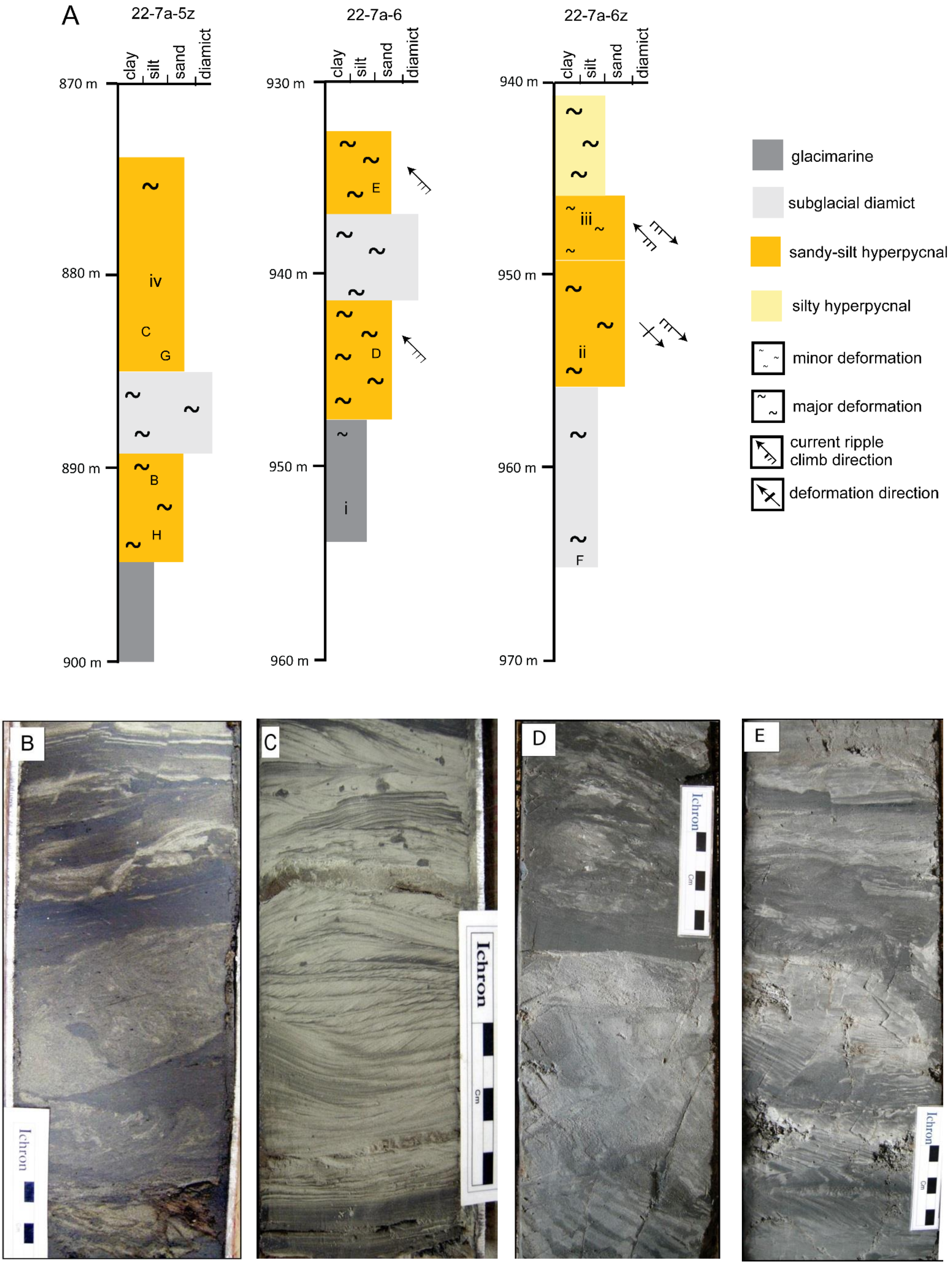
A
22-7a-5z
22-7a-6
22-7a-6z
clay
silt
sand
diamict
870 m
880 m
890 m
900 m
930 m
940 m
950 m
960 m
940 m
950 m
960 m
970 m
iv
C
G
B
H
E
D
i
iii
ii
F
glacimarine
subglacial diamict
sandy-silt hyperpycnal
silty hyperpycnal
minor deformation
major deformation
current ripple climb direction
deformation direction
B
C
D
E
Ichron

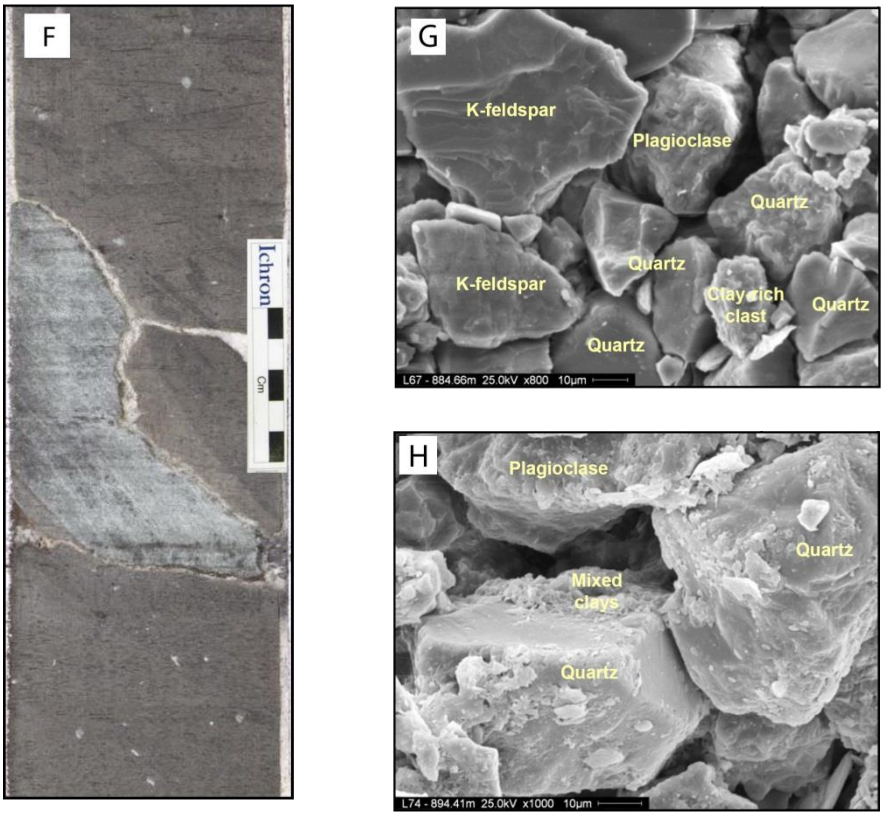

fig. S4. Core stratigraphy, images, and interpretation. (**A**) Core logs, sedimentology and kinematics from the three Aviat cores. Directional indicators, ripple climb and fold vergence, point respectively in the direction of flow/compression with north to the top. Water flow directional indicators (ripple climb direction) are equivocal, suggesting discharge directions coming from the south-east and north-west quadrants. This likely reflects the complexity of the bed topography over which the traction currents were flowing and the influence of eddies. Deformation kinematics, south-east quadrant verging kink bands, suggest ice flow from the north-west quadrant, which probably represents control of the local topography on the compression direction. The overall geometry of the fan suggests ice flow and sediment flux from the SW to NE. Letters indicate the location of B–H in this Figure. Roman numerals in circles, i–iv, show the location of images/samples in Fig. 5A–D, respectively. (**B**) Major post deposition deformation, showing multiple styles. (**C**) Mixed silt and very fine sand laminae showing gradational vertical and lateral changes in cross-lamination and particle size, with erosion surfaces and rip-up clasts apparent. (**D**) Thin bedded lower sandy-silts showing both brittle and ductile post deposition deformation, overlain by pervasively deformed silts. (**E**) Rippled very fine sand to silt deposited form low-concentration flows showing brittle and ductile post deposition deformation. Note reverse faults in a

number of locations. (**F**) Pervasively deformed silty-clay subglacial diamict containing a large angular out-sized clast of mica schist. (**G**) SEM micrograph illustrating the freshness of the grains, noting the lack of solution features or overgrowths. (**H**) SEM micrograph illustrating the freshness of the grains.

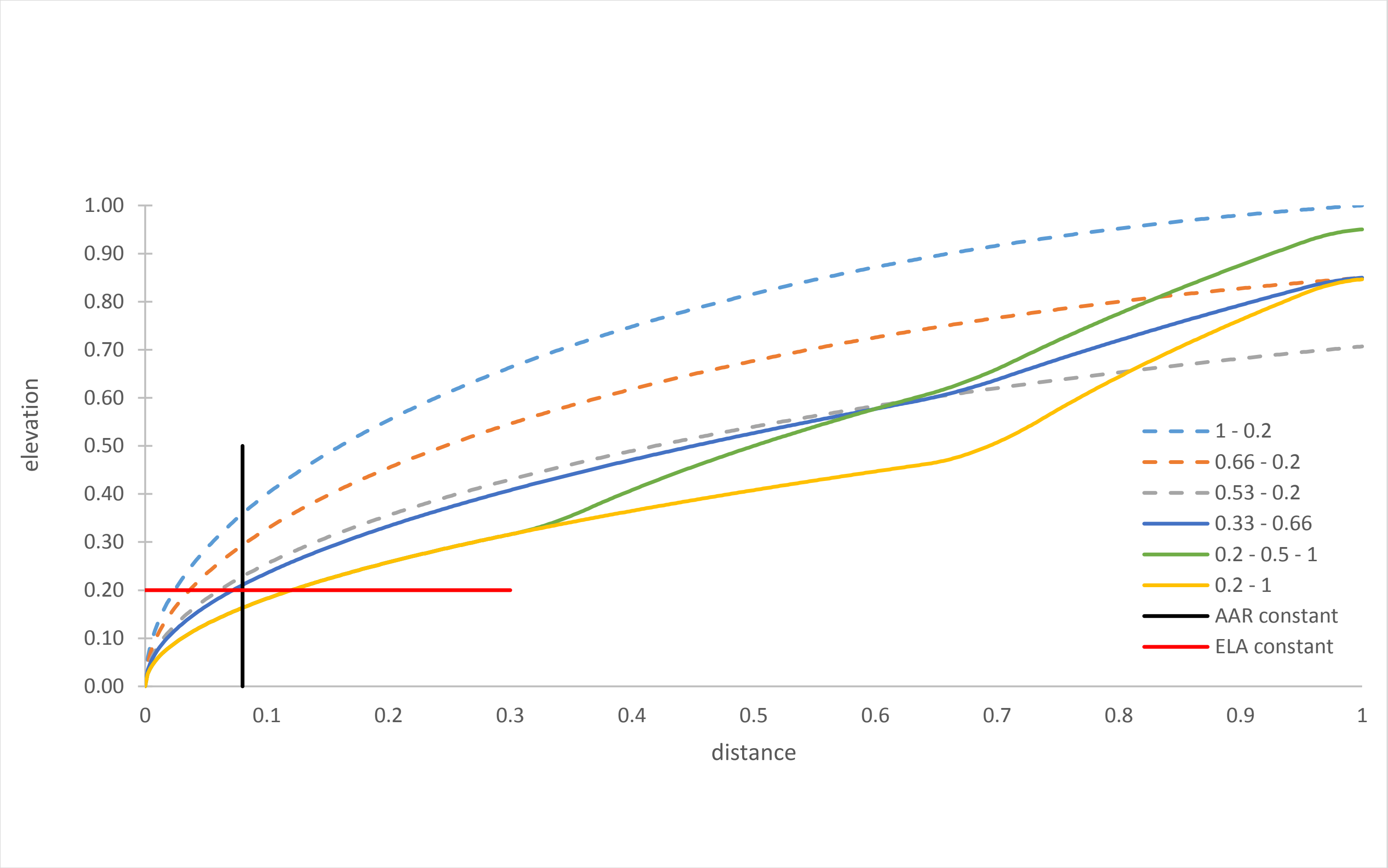

fig. S5. Theoretical ice sheet surface profiles demonstrating the effects of basal resistance and topography. The dashed lines indicate ice sheets with basal resistance/shear stress decreasing (linearly) from the ice margin (0) to the ice divide (1). Lower elevations result from lower initial conditions (a basal resistance of 1 is equivalent to 150 kPa) and represent increasing slipperiness of the bed. The solid lines indicate the varying effects of increasing basal resistance generated by a rough-to-ice-flow fluvial landscape. The three zones represent an idealisation of a transect from the ice divide to the ice margin of an Early Pleistocene ice sheet originating in the Scottish mountains and flowing east into the North Sea (the effects of calving are ignored). The implications for mass balance are indicated for a constant ELA (horizontal red line) such that the accumulation area ratio (AAR) changes or for a constant AAR (vertical black line) where the ELA will be lowered for successively thinner ice sheet configurations. The ice surface equilibrium profiles were calculated using an exact solution for the perfect plasticity assumption for the flow of ice (*69*) solved numerically (*70*).

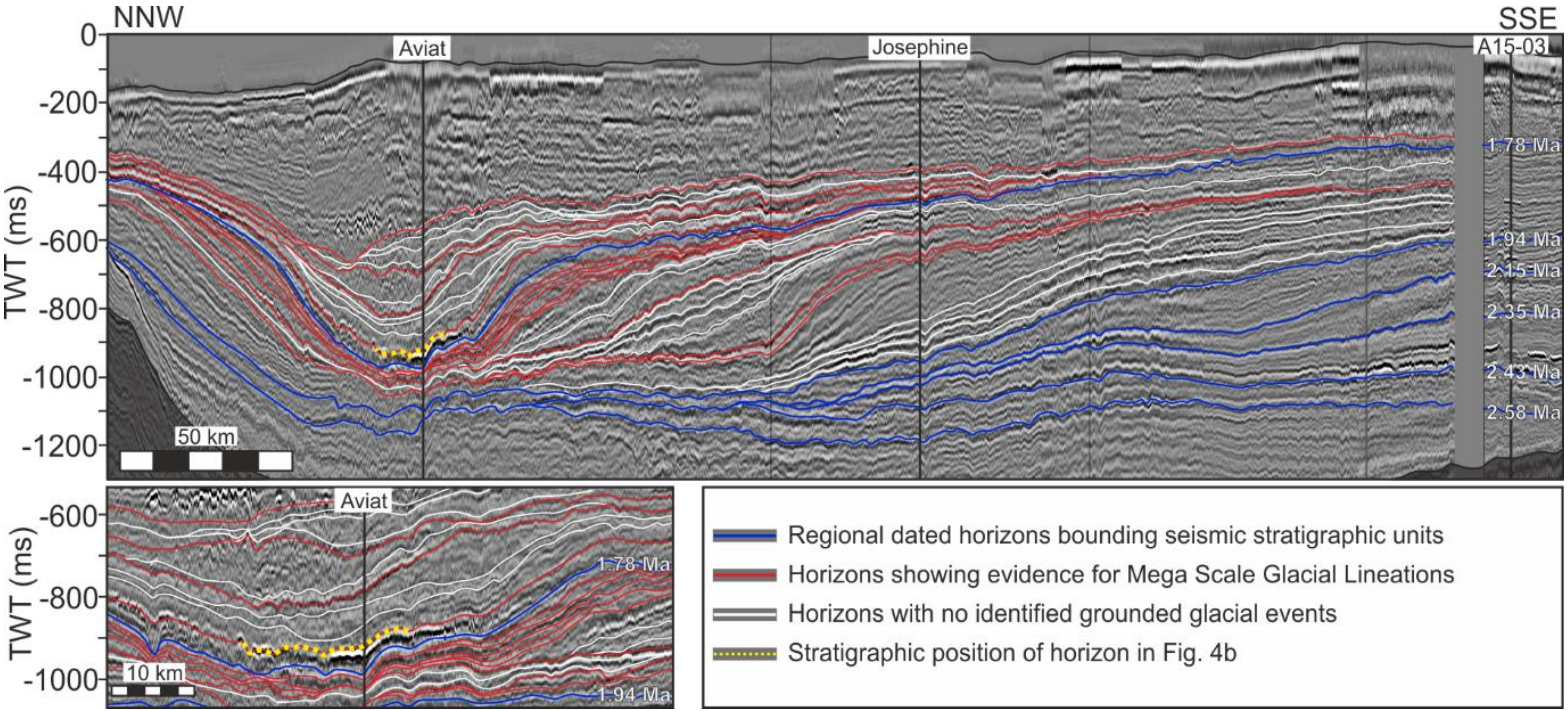

fig. S6. A section showing the basis for the seismic stratigraphic framework tied to the magnetic reversal and palynology-based chronology from A15-03 in the Dutch sector of the North Sea (blue lines), which is corroborated by ages from Josephine and Aviat in the UK sector (*16*). This allowed a robust chronology to be established for the basin scale mapping. Horizons with clear evidence for grounded ice (mega scale glacial lineations) are shown in red and those lacking evidence for grounded ice are shown in white. The dashed yellow line indicates the location of the horizon shown in Fig. 4B taken from the centre of the basin. MegaSurvey 3D seismic data curtesy of PGS.

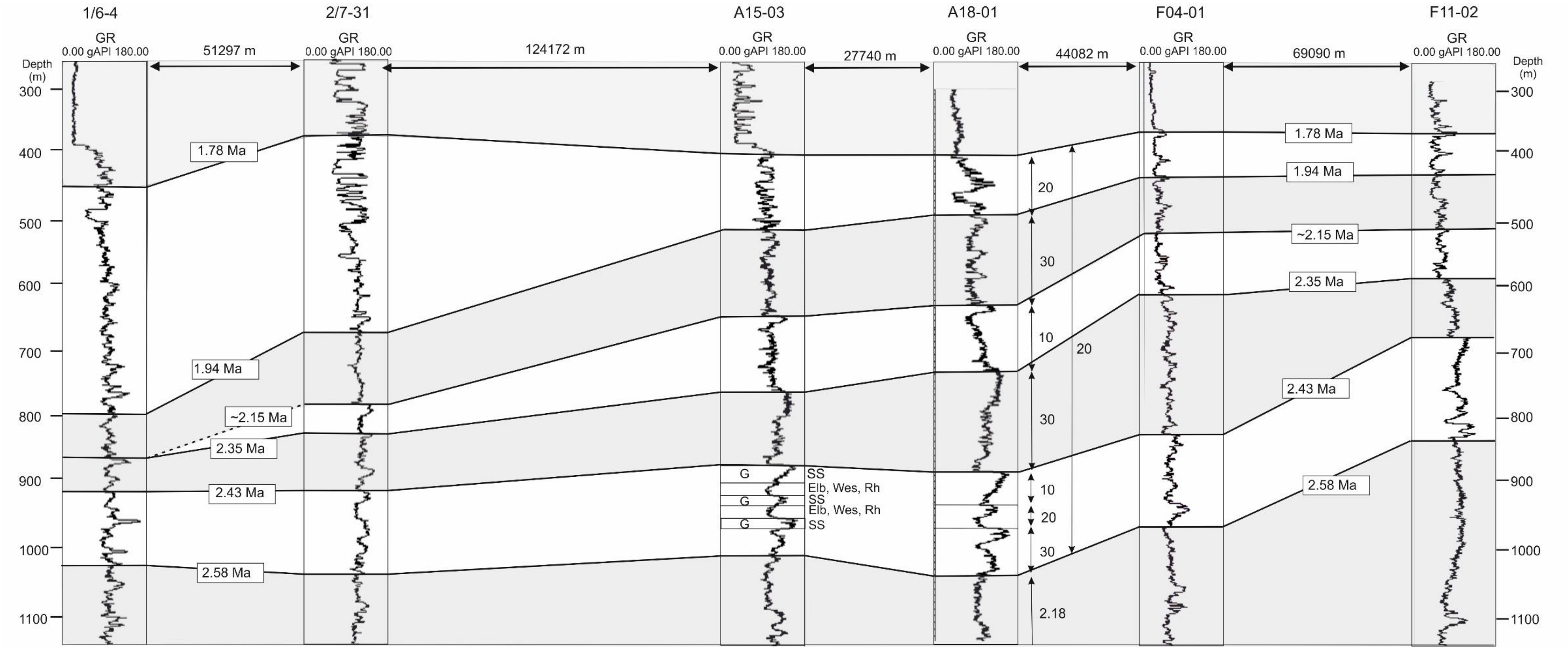

fig. S7. Downhole gamma ray logs from the Dutch sector of the southern North Sea. The age of key stratigraphic horizons are highlighted. Glacial cycles show an increasing gamma ray signal as the glacial stage progresses, related to high sedimentation rates during the deglaciation. Interglacials appear to have a lower delivery of clay-rich sediments into the basin. These early glaciations are characterised by an overall higher mean gamma ray signal which subsequently declines back to a lower baseline, near equivalent to the pre-Pleistocene mean. The changing gamma ray signal is interpreted to reflect the initial stripping of clay-rich regolith from the terrestrial landmasses of Europe, with the regolith supply slowly being exhausted as time and erosion progresses. Provenance data from A15-03 comes from (*71*). The sediment accumulation rates (vertical arrows with numbers) are in km^3 per 1000 years for the Southern North Sea and come from (*17*). G represents a glaciation, SS is provenance from the Scandinavian shield, while Eib, Wes and Ph are provenance from the Elbe, Weser and Rhine catchments, from (*71*).

table S1. Details for sites where Early Pleistocene iceberg scours have previously been identified. Site locations are shown in fig. S1, depths are in two way travel time (ms) (* the depth for site 5 is in metres below seafloor), ages are those assigned to the sites by the authors of the studies and the source for the information, where the number equates to the reference list in this paper.

**site**	**depth ms TWT of iceberg scours**	**age Ma**	**source**
1	660	2–2.2	(*15*)
2	600	> 1.9	(*66*)
3	1020	2.6–0.5	(*18*)
4	800	1.7	(*67*)
5*	> 800 m	2.6–1.2	(*68*)
6	1060		(*8*)
7	600–1000	<2.4	(*8*)

table S2. Summary information for the biostratigraphical evaluation of Aviat cores 22-7a-5z and 22-7a-6z. The position of the Pliocene/Pleistocene boundary is difficult to precisely identify and has been determined at 889 m in 22-7a-5z and 952.7m in 22-7a-6z. Several age diagnostic taxa are recorded at about this boundary, but due to the elevated reworked fraction of species ranging from the Carboniferous to Miocene and Early Pliocene it is difficult to be precise. The dating resolution here is similar to all such data from the North Sea as there are simply a lack of biostratigraphic datums with which to further constrain ages (*15*). (**A**) Five palynological events are correlated between the two wells but the sparse occurrence (isolated and single on occasion) and potential for reworking make boundary identification difficult. Events *Imu* and *Selo* are diagnostic for the Pleistocene while *Ita* ranges from Pleistocene and older. The top occurrence *Amu* suggests that this section is not younger than the Gelasian Stage. Events *Bpli, Ila* and *Ptu* are indicative of the Pliocene epoch. The Early Pleistocene is distinguished by the occurrence of *Spiniferites elongates* and the abundant occurrence of the *Bitectatodinium* Group (952.7 m in 22-7a-6z) which has previously been recorded at the base of the Pleistocene (*15*) and also the presence of *Amiculosphaera umbraculum* (943 m in 22-7a-6z) suggests that this section is not younger than the Gelasian Stage. (**B**) Six micropalaeontological zones have been identified indicating a number of micropalaeontological events common to both the wells. TNS – top not seen.

A

**Key**	**Palynological Event**	**Well 22/7a-5Z**	**Well 22/7a-6Z**
Ica	Questionable (base?) occurrence of *Impagidinium cantabrigiense*	Not present	942.4 m
Imu	Single (base?) occurrence *Impagidinium multiplexum*	884.37 m	947.9 m
Ita	Single (top?) occurrence *Invertocysta tabulata*	890.8 m	Not present
Selo	(base?) occurrences of *Spiniferites elongatus*	890.8 m	952.45 m
Bpli	Possible in situ TO *Barssidinium pliocenicum*	892.14 m	952.45 m
Ila	Single occurrence *Invertocysta lacrymosa*	892.14 m	955.1 m

Ptu	Single occurrence *Pyxidinopsis tuberculata*	892.75 m	953.3 m
Aum	Top occurrence *Amiculosphaera umbraculum*	720 m	943 m

B

**Zone**	**Faunal character**	**Well 22/7a-5-5Z**	**Well 22/7a-6Z**
6	Low diversity, dominated by *C. laevigata* and *E. excavatum*	720 m (TNS)	
5	Increase diversity & numbers, with *C. grossus* and *T. fluens*	811 m	
4	Sparse recovery	847 m	942.4 m (TNS)
3.2	Re-appearance abundant *E. excavatum*	871 m	946.4 m
3.1	Appearance reworked Late Cretaceous planktonics	880 m	947.35 m
2	Moderate to high diversity, low dominance, nodosariids	890.22 m	951.1 m
1	Increase in relative abundance of *C. laevigata*, reduced diversity	910 m	961 m
